# Towards Portable MEMS Oscillators for Sensing Nanoparticles

**DOI:** 10.3390/s22155485

**Published:** 2022-07-22

**Authors:** Malar Chellasivalingam, Arthur T. Zielinski, Thomas S. Whitney, Adam M. Boies, Ashwin A. Seshia

**Affiliations:** 1 Department of Engineering, University of Cambridge, Cambridge CB2 1PZ, UK; tsw33@cam.ac.uk (T.S.W.); amb233@cam.ac.uk (A.M.B.); 2 The Nanoscience Centre, University of Cambridge, Cambridge CB3 0FF, UK; 3 Department of Chemistry, University of Cambridge, Cambridge CB2 1EW, UK; arthurtzielinski@gmail.com; 4 Centre for Atmospheric Science, University of Cambridge, Cambridge CB2 1EZ, UK

**Keywords:** MEMS, oscillators, sensors, resonant frequency, nanoparticles, particulate matter, resonators, indoor particles, silver nanoparticles

## Abstract

This paper reports on the design, and implementation of piezoelectric-on-silicon MEMS resonators installed within a portable experimental setup for sensing nanoparticles in a laboratory environment. MEMS oscillators with a center frequency of approximately 5.999 MHz are employed for sensing 50 nm size-selected silver nanoparticles generated in the laboratory. The same experimental setup is then assembled to sense indoor particles that are present in the laboratory environment. The challenges associated with particle deposition as a result of assembling the portable experimental setup is highlighted. Furthermore, the MEMS oscillators demonstrate that the total mass of silver nanoparticles deposited onto the MEMS resonator surface using the inertial impaction technique-based experimental setup is approximately 7.993 nanograms. The total indoor particle mass accumulated on the MEMS resonator surface is estimated to be approximately 1.732 nanograms and 26.9 picograms for two different runs. The frequency resolution of the MEMS oscillator is estimated to be approximately 32 ppb and, consequently, the minimum detectable particle mass is approximately 60 femtograms for a 9.2 s integration time.

## 1. Introduction

The advent of miniaturized sensors integrated into wired or wireless sensor networks has made it possible to monitor environmental parameters continuously and with high fidelity [1,2]. One such area of interest where these sensors can overcome the limitations associated with current monitoring instruments is gravimetric sensing. While a range of technologies have been developed for gravimetric sensing, several of these are limited by their size, portability, cost, power consumption and inability to measure over a range of analytes of interest. Microelectromechanical systems (MEMS) and CMOS technologies provide a promising integration platform [3] in this context, enabling the miniaturization and integration of gravimetric sensors for gas analysis and particulate monitoring while addressing a number of the limitations associated with current technologies [4].

An application scenario in the context of gravimetric sensing where MEMS sensors can provide an added advantage is in the sensing of ultrafine particles that are less than 100 nanometers in diameter. The significance of sensing ultrafine particles [5,6,7] has been widely recognized due to their detrimental effects on human health, as discussed in [8,9,10,11,12,13,14,15,16,17]. Current approaches include commercial condensation particle counters, but these provide an estimate of number concentration rather than a direct measure of total mass or mass concentration and require a system to enlarge the particles to a sufficiently large diameter through vapor condensation prior to detection. Additionally, once particulates are collected onto the surface of a MEMS sensor, other techniques can be applied for further characterization including compositional analysis [18]. This paper demonstrates the applicability of MEMS sensors, specifically piezoelectric-on-silicon resonant mass sensors, for detecting ultrafine particles following on previous work [19] applied to two use cases viz. (1) to characterize silver nanoparticles generated in a laboratory setup and (2) to the detection of particulate matter in an indoor environment.

An additional feature of MEMS sensors that is beneficial for this purpose is the potential for miniaturization and integration, including fabrication compatibility with standard Complementary Metal Oxide Semiconductor (CMOS) Very Large Scale Integration (VLSI) processes [20]. Specifically, MEMS oscillators have been previously demonstrated as critical building blocks for several types of sensors, including mass sensors, biological sensors that detect molecular interactions, electrometers, accelerometers, AFM probes, and pressure sensors. In all these transducers, the performance of the MEMS oscillator is crucial, and is frequently a limiting factor in the overall system performance [21]. It should be noted that piezoelectric-on-silicon MEMS resonators have emerged as a promising candidate as building blocks for oscillators and sensors combining the benefits of good electromechanical coupling and improved power handling under ambient conditions relative to equivalent capacitive MEMS devices [22,23,24]. These features consequently enable improvements in terms of phase noise and frequency stability of the oscillator implementation that integrates the piezoelectric-on-silicon resonator as the frequency determining element [25]. Leveraging these benefits of piezoelectric transduction, several papers in the literature have demonstrated applications to gravimetric sensing including to particulate matter detection and mass sensing in liquids [26,27,28,29,30,31,32,33,34,35,36,37,38,39,40,41,42,43,44,45,46,47,48,49,50,51,52,53,54,55,56,57,58].

In this paper, we report on piezoelectrically transduced, bulk acoustic MEMS resonators embedded in a phase-locked oscillator loop to detect ultrafine particles such as the silver nanoparticles generated in the laboratory and particles observed in an indoor environment. The focus of this paper is on validating the performance of such sensors for sensing nanoparticles, either in a natural indoor setting or those created in a controlled laboratory process. The devices operate by monitoring the output frequency shift of the MEMS oscillators due to ultrafine particles adsorbing onto the resonator surface.

## 2. Materials and Methods

**Figure 1:**
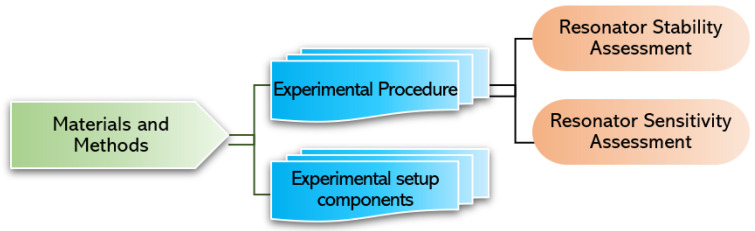
Organization structure of the materials and methods section.

This section is divided into two parts, as shown in Figure 1: first, the different components assembled to construct the experimental setup required to conduct silver nanoparticle deposition and indoor particle deposition, and thereby to characterize the MEMS resonators, are detailed. Second, the experimental procedure for conducting a sensitivity and stability assessment of the MEMS resonators is outlined.

### 2.1. Experimental Setup Components

This paper details two types of experimental setup—(i) an indoor particle real-time monitoring setup and (ii) a silver nanoparticle real-time monitoring setup. In particular, two types of indoor particle deposition are performed, and, in both cases, the indoor particles present in the room atmosphere are not size selected. The difference between the two types of indoor particle deposition lies in that the first set of indoor particle deposition has a T-shaped tubing arrangement, and such tubing arrangement is removed in the second case, to assess the impact of tubing arrangement on the deposition of particles onto the MEMS resonator surface.

**Figure 2:**
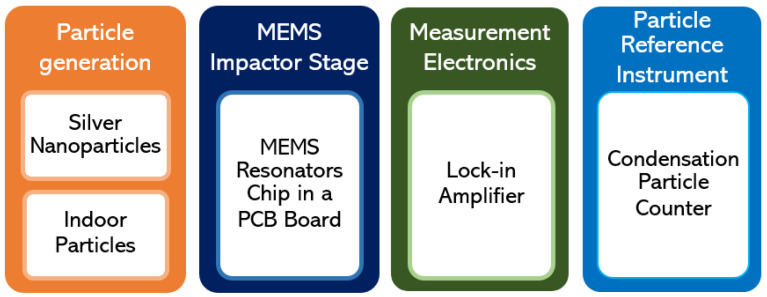
Different components of the experimental setup used in this study.

Figure 2 depicts the different components assembled to construct the experimental setup required to characterize the MEMS resonators and conduct particle deposition on the resonator surface.

#### 2.1.1. MEMS Impactor Stage (MIS)

The MEMS resonator is positioned within the MEMS Impactor Stage (MIS) and particle deposition is performed on the resonator surface based on the principle of inertial impaction. In this technique, particles in air flow greater than a certain size will pass through a nozzle inlet to reach the surface of the MEMS resonator acting as the impaction plate. A vacuum pump is connected at the MIS exhaust in order to draw the particles towards the MEMS resonator surface through the nozzle inlet. More details regarding the functioning of the MIS can be found in [7,19,59].

**Figure 3:**
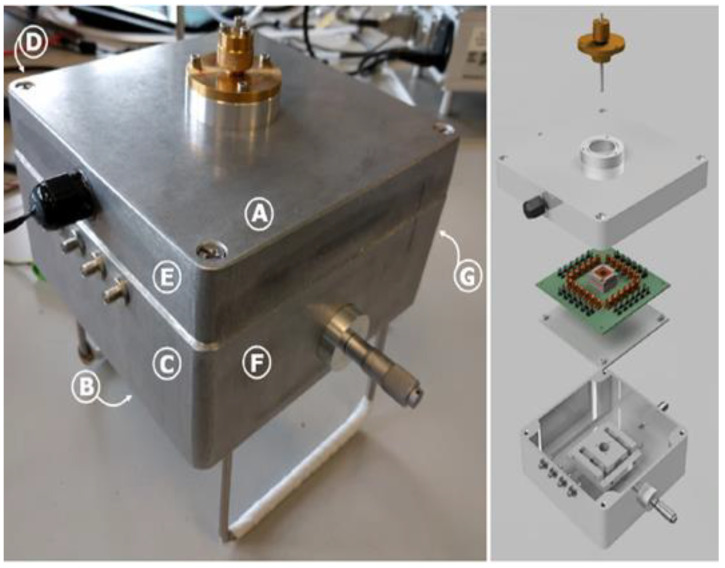
(**Left**) MEMS Impactor Stage and (**Right**) exploded view of the MEMS Impactor Stage [26].

Figure 3 depicts the MEMS Impactor Stage (MIS) used in particle deposition experiments; Figure 3 (left) shows the MEMS Impactor Stage in its sealed position, and Figure 3 (right) shows an exploded view of the MEMS Impactor Stage, with the letters (A–G) corresponding to the location of the inlet, outlet, signal port, redundant port, temperature/RH port, stage alignment control, and camera viewports, respectively. The key parameters of the MEMS Impactor Stage affecting particle deposition on the MEMS resonator surface are the nozzle jet diameter, impaction plate dimensions, throat length of the nozzle, distance between the nozzle throat length and the impactor plate. The MEMS Impactor Stage described herein is designed such that the cut-off diameter indicating 50% collection efficiency is small enough to allow sufficient deposition by impaction [60].

#### 2.1.2. MEMS Resonators Design, Fabrication and Transduction

The MEMS resonator used in this experiment is a square plate with a side length of 200 µm and a silicon device layer thickness of 10 µm. The square-plate resonator is fixed at its two corners by T-shaped anchor beams. An AlN layer is deposited over the silicon device layer to enable piezoelectric transduction. Metal electrodes are patterned on top of the piezoelectric material for driving the resonator and sensing the motional response. Interconnects to these electrodes are routed over the connecting tether supports.

**Figure 4:**
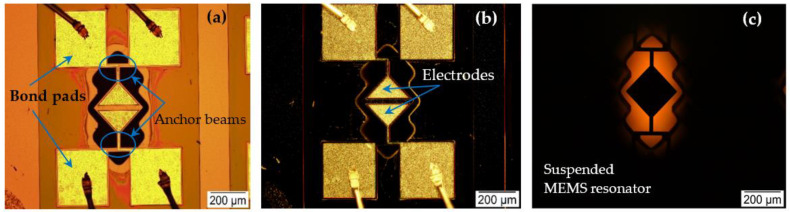
(**a**) Bright-field and (**b**,**c**) dark-field microscopic images of the microfabricated 200 µm side-length square-plate MEMS resonator suspended by T-shaped anchor beams.

An ac voltage is applied through the drive electrode to excite the resonator into motion. As a result, a time-varying force is applied to the resonator, with the applied forcing frequency equal to the frequency of the applied ac signal. The output electrical signal is detected by a sense electrode patterned on the same substrate, as shown in Figure 4.

The designed MEMS resonator is fabricated by MEMSCAP Inc., USA in a commercial foundry using a silicon-on-insulator MEMS process [61]. The MEMS die is packaged in a chip carrier, attached to the PCB board, and installed within the MIS. As previously illustrated in Figure 3, the electrical connections to the PCB board are established through the inlet and outlet ports of the MIS and the dimensions of the MEMS resonators are outlined in Table 1.

#### 2.1.3. Resonator Equivalent Circuit Model

**Figure 5:**
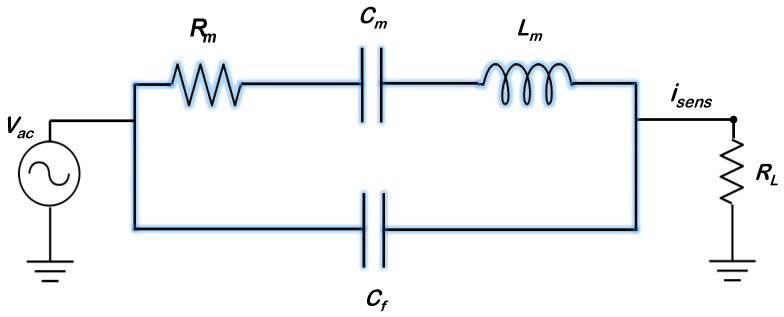
Equivalent electrical circuit representation of the MEMS resonator.

The MEMS resonator can be described by an electrical equivalent consisting of a series LCR circuit [62], as shown in Figure 5.

In Figure 5, $R_{m}$ represents the motional resistance, $C_{m}$ represents the motional capacitance, $L_{m}$ represents the motional inductance, and $C_{f}$ is the feedthrough capacitance between drive and sense electrodes. The parasitic feedthrough may have substrate coupling sources and couples the drive voltage over to the motional current sensing port. The MEMS resonator modelled as a series LCR circuit has a series resonance ($\omega_{m}$) defined by the following equation:

(1) $$\omega_{m}={\left(L_{m}C_{m}\right)}^{-1/2}$$

A phase-locked loop to achieve accurate resonant frequency tracking is accomplished using the HF2LI lock-in amplifier (Zurich Instruments). High-resolution measurements are possible using this frequency tracking approach.

**Figure 6:**
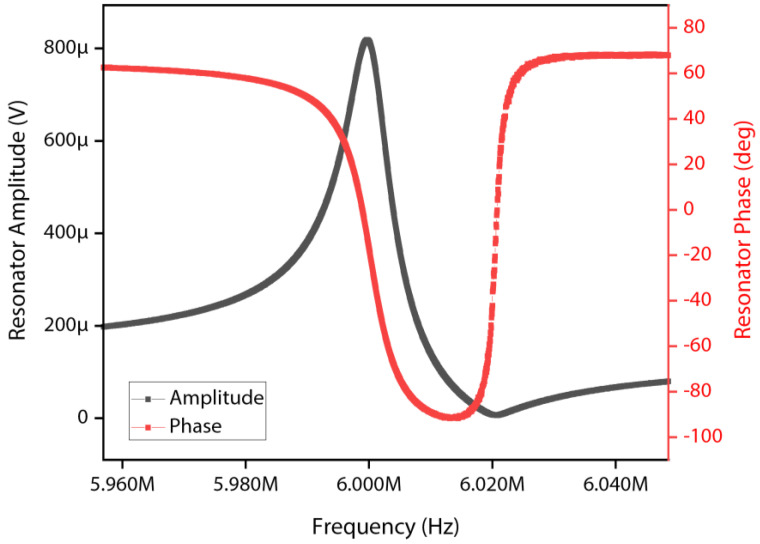
Measured open−loop frequency sweep transmission response of the MEMS resonator.

Measurements of the equivalent series LCR circuit parameters can also be extracted from the open-loop frequency sweep response recorded by the HF2LI lock-in amplifier. Figure 6 depicts the measured open-loop frequency sweep response of the MEMS resonator described in this work.

The linear equivalent circuit model parameters extracted from open-loop frequency sweep measurement observed in Figure 6 are summarized in Table 2.

#### 2.1.4. Indoor Particles Experimental Setup

**Figure 7:**
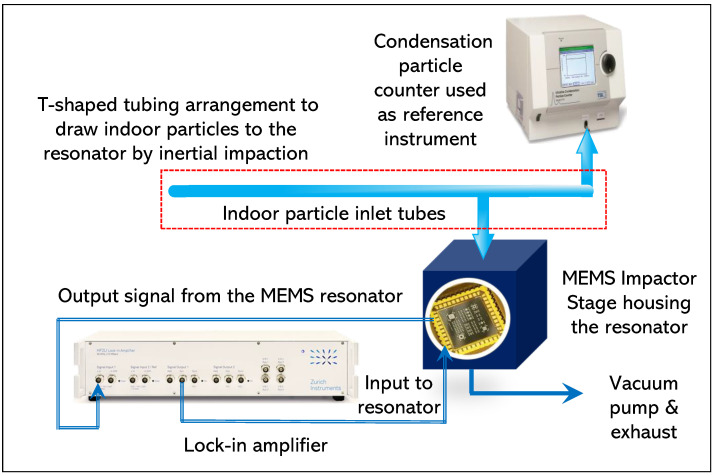
Experimental setup for drawing indoor particles towards MEMS resonators through inertial impaction. A T-shaped tubing arrangement split a common inlet—one to the CPC and the other to the MEMS Impactor Stage nozzle.

As mentioned previously, two types of indoor particle deposition are performed–with and without T-shaped tubing arrangement. The first type of indoor particle deposition involved a T-shaped tubing arrangement, as shown in Figure 7. The T-shaped tubing arrangement connects the particle reference instrument, the condensation particle counter, and the MEMS Impactor Stage in parallel through a common inlet. The MEMS Impactor Stage measures the mass of the particles entering at its inlet and which gets deposited on the resonator surface through inertial impaction mechanism. The condensation particle counter (CPC) measures the particle number concentration entering at its inlet, providing a reference instrument for comparison.

**Figure 8:**
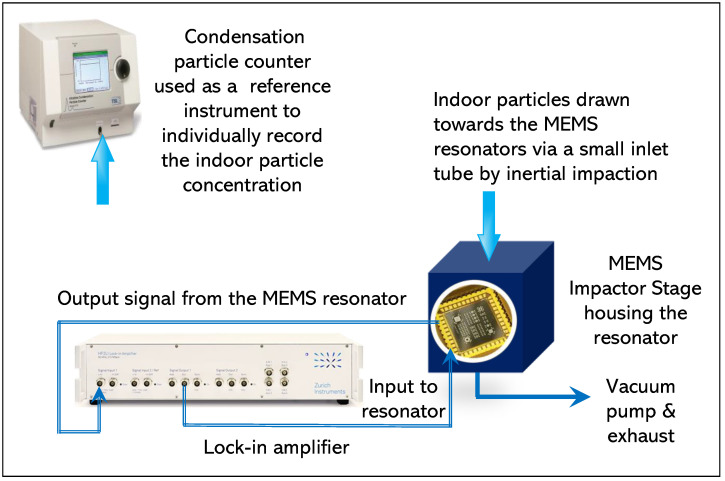
Experimental setup for drawing indoor particles towards MEMS resonators through inertial impaction. CPC and the MEMS resonator monitored indoor particles independently.

The second set of indoor particle deposition involved removing the T-shaped tubing arrangement from the experimental setup demonstrated in Figure 7 and is illustrated in Figure 8. Since the MEMS Impactor Stage is composed of a nozzle inlet, a small inlet tube protruding from the nozzle inlet is used for drawing particles towards the MEMS resonator based on inertial impaction technique. The diameter of the single jet nozzle used in this experiment is approximately 0.14 mm and the set flow rate is 0.6 L/min.

**Figure 9:**
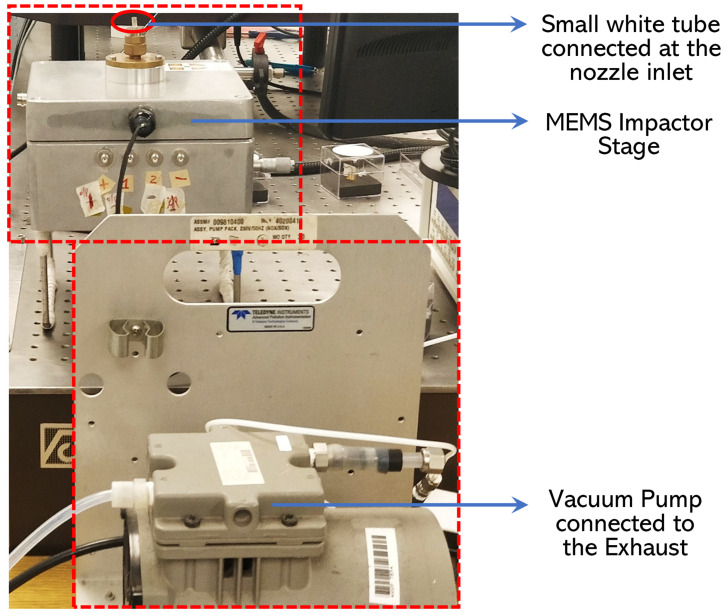
Laboratory experimental setup without T-shaped tubing arrangement to draw indoor particles towards the MEMS resonator.

Therefore, in this experimental setup, individual tubes are connected to the condensation particle counter (CPC) and the MEMS Impactor Stage (MIS) setup, respectively, as shown in Figure 8. Furthermore, Figure 9 depicts the experimental setup for the second set of indoor particle deposition as seen in the laboratory.

#### 2.1.5. Silver Nanoparticles Generation Experimental Setup

**Figure 10:**
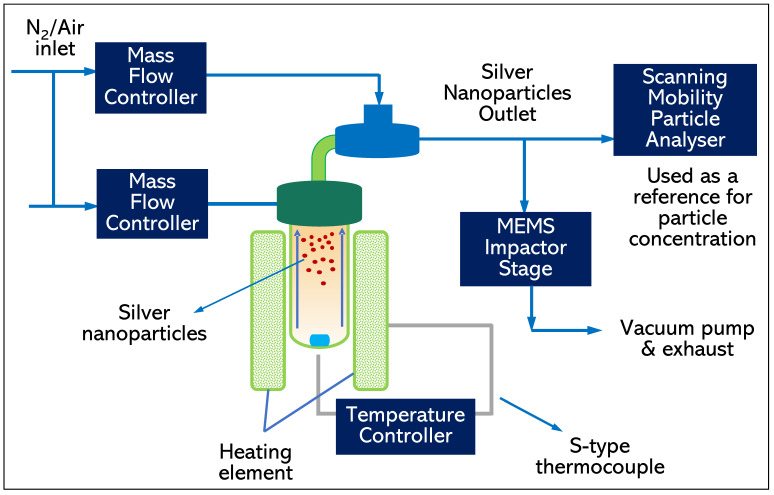
Experimental setup interfacing the source of silver nanoparticles to the MEMS Impactor Stage for deposition on MEMS resonator surface by inertial impaction.

The silver nanoparticle generator has a glass test tube arrangement as depicted in Figure 10, used to produce silver nanoparticles in the laboratory [63]. Nitrogen gas is passed onto a small piece of silver (Ag) placed at the bottom of the glass test tube. The glass test tube is then heated by a ceramic heater arrangement governed by a PID temperature controller, while an insulator arrangement enclosed the test tube. Due to the applied heat, the silver nanoparticle fumes generated inside the test tube, containing the nanoparticles is made to pass to the outlet of the test tube. A Scanning Mobility Particle Analyzer (SMPS) connected in parallel with the MEMS Impactor Stage was connected to the common outlet of the glass test tube. The size distribution of the generated silver nanoparticles was recorded using the Scanning Mobility Particle Analyzer, and the particle size was recorded to be approximately 50 nm. Since the generated silver nanoparticle concentration was too high (in the order of #$10^{7}$ particles/${\mathrm{cm}}^{3}$) to be recorded by the condensation particle counter (CPC 3025A) used in this experiment, the generated particles were simply passed onto the MEMS Impactor Stage inlet. Figure 10 describes an outline of the experimental setup used for silver nanoparticle deposition experiment.

### 2.2. Experimental Procedure

The experiment in this research study is primarily concerned with depositing both laboratory-generated silver nanoparticles and indoor particles observed in a room atmosphere onto the surface of the MEMS resonator. A HF2LI lock-in amplifier was used to determine the mass sensitivity of the MEMS resonator to the deposited nanoparticles. This section details the experimental procedure used to deposit silver nanoparticles and indoor particles onto the MEMS resonator surface.

#### 2.2.1. The Silver Nanoparticle Deposition Experiment

The experiment began with the collection of 50 nm silver nanoparticles onto the 200 µm side-length square-plate MEMS resonator positioned within the MEMS Impactor Stage (MIS) setup. Using a single jet nozzle with a diameter of approximately 0.14 mm and a flow rate of 0.6 L/min, the vacuum pump was used to draw the nanoparticles towards the MEMS resonator surface through inertial impaction. The particle deposition procedure onto the MEMS resonator is described in detail in [7]. The silver nanoparticle deposition experiments were carried out using a T-shaped tubing arrangement in which the silver nanoparticles drawn through a common inlet were split into two ends. The CPC inlet, which functioned as the reference instrument, was connected to one end of the tube, and the MEMS Impactor Stage was connected to the other, allowing the CPC to simultaneously monitor the silver nanoparticles deposited on the MEMS resonator surface. However, as previously stated in Section 2.1.5, the CPC (3025A) did not read the exact concentration of silver nanoparticles generated during the experiment because the particle concentration exceeded its inherent detection limit. By establishing a closed-loop using the PLL function in the HF2LI lock-in amplifier, real-time deposition of the 50 nm silver nanoparticles onto the MEMS resonator surface was monitored.

The open-loop frequency response of the MEMS resonator was measured after certain time intervals of silver nanoparticle deposition on its surface. Although the monitoring of silver nanoparticle deposition was intended to be continuous, there were some unforeseen interruptions of the resonance tracking in the HF2LI during particle deposition resulting in a piece-wise compilation of the datasets over certain time periods.

**Figure 11:**
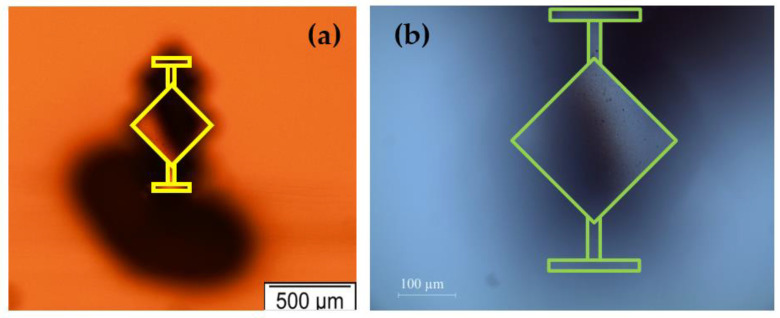
(**a**,**b**) Bright-field microscopic images demonstrating the deposition of silver nanoparticles on the backside surface of 200 µm side-length square-plate MEMS resonator.

**Figure 12:**
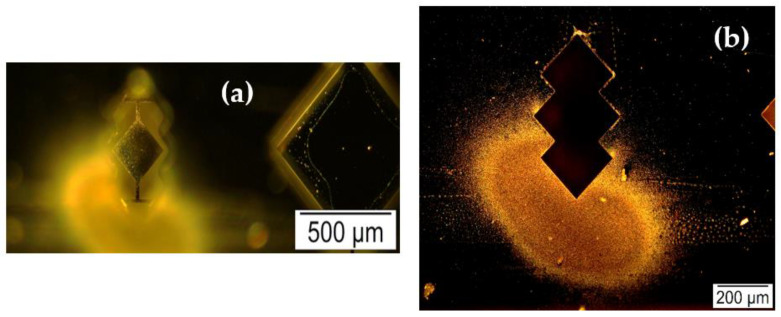
Darkfield microscopic images of silver nanoparticles deposited onto (**a**) the MEMS resonator surface and (**b**) those that deposited outside the resonator surface, respectively.

Figure 11 and Figure 12 depict the silver nanoparticles deposited onto the MEMS resonator surface during this experiment. The silver nanoparticles are seen deposited both inside and outside the resonator surface during the experiment given the relatively small dimensions of the resonator compared to the rest of the arrangement [7]. However, only the silver nanoparticles collected on top of the resonator surface are considered for analysis.

#### 2.2.2. The Indoor Particle Deposition Experiment

**Figure 13:**
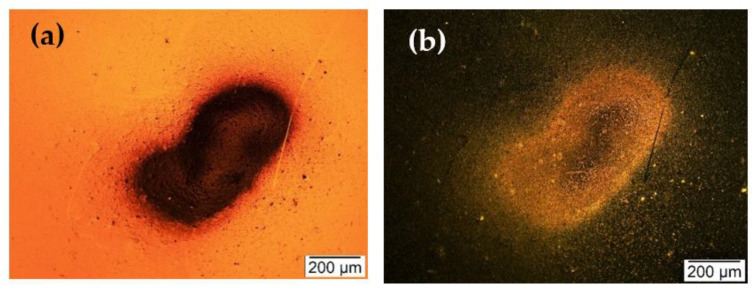
(**a**) Bright field and (**b**) dark-field microscopic images of the indoor particles deposited on a blank silicon substrate prior to the deposition on the MEMS resonator.

As explained in Section 2.1.4, since the first set of indoor particle deposition required a T-shaped tubing arrangement, the indoor particles were first collected on a blank silicon substrate for approximately 3 h. This deposition is to ensure that only indoor particles were collected on the resonator surface and to extract any other type of particles stuck to the walls of the tube from previous particle deposition experiments [5,6,7]. In this experiment, the indoor particles were not size selected, and the bright-field and dark-field images of indoor particles collected on a blank silicon substrate are shown in Figure 13 as microscopic images.

Following the deposition on a blank silicon substrate, the indoor particles were collected onto the MEMS resonator with a side length of 200 µm, and the resonant frequency shift was tracked continuously. In addition, the open-loop frequency response of the MEMS resonator was recorded prior to deposition on the MEMS resonator using the HF2LI lock-in amplifier as described in Section 2.1.3.

The first set of indoor particle deposition experiment began by depositing indoor particles observed in the room atmosphere onto the MEMS resonator surface for approximately 2.6 h. Initially, the vacuum pump was not turned on to draw the particles towards the resonator surface, and the CPC monitored the indoor particle concentration independently. This step was carried out to determine whether the particle concentration was stable enough for the indoor particles to be deposited on the MEMS resonator surface. When the indoor particle concentration measured by the CPC stabilized after initial 1.5 h, the vacuum pump was activated, and the indoor particles were drawn towards the MEMS resonator surface.

In this first set of indoor particle deposition experiment, the indoor particle monitoring with the MEMS resonator began at time t = 0 and lasted initially for 18.65 min. The experiment was interrupted for approximately 2 min due to the HF2LI lock-in amplifier losing lock. Once this was rectified, the indoor particle monitoring continued at time t = 20 min until time t = 122 min. After this period, the vacuum pump was turned off for approximately 2 min, and again the MEMS resonator continued measuring indoor particle mass from time t = 124 min to time t = 138.5 min. Following this period, the experiment was halted for approximately 3 min, during which time the vacuum pump was turned off. The experiment was then repeated at time t = 141 min until it was terminated. Therefore, in this first set of indoor particle deposition experiments, the data collected from time t = 0 min until time t = 156 min were analyzed.

**Figure 14:**
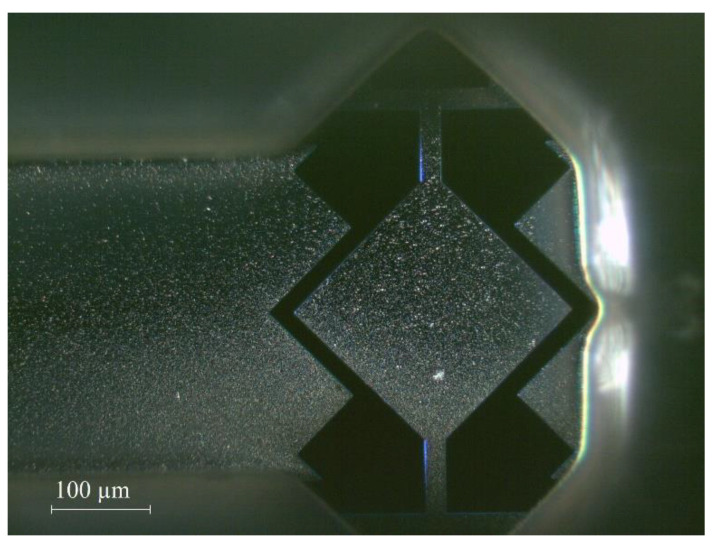
Indoor particles deposited on the MEMS resonator surface by inertial impaction.

Figure 14 depicts the indoor particles deposited on the 200 µm side-length square-plate MEMS resonator in the first type of indoor particle deposition experiment. It should be noted that the indoor particles were not size selected for the experimental work reported in this study.

The second type of indoor particle deposition experiments was performed on the MEMS resonator for approximately 2 h continuously, similar to the first set, and the resonant frequency was tracked in real time. The goals of this experiment are twofold: (1) to obtain the deposition of indoor particles on the surface of the MEMS resonator without the use of a T-shaped tubing arrangement and, as a result, to determine the impact of the tubing arrangement on particle deposition, and (2) to continuously monitor the resonant frequency shift data with few time intervals between measurements. Accordingly, the CPC and the MEMS resonator read the indoor particle concentration independently in the second set of indoor particle deposition experiments.

#### 2.2.3. The Frequency Stability Experiment

Long-term frequency stability measurements for the 200 µm side-length square-plate MEMS resonator are obtained by monitoring the resonant frequency by establishing a phase-locked loop for the desired mode occurring at 5.999 MHz using the HF2LI lock-in amplifier. The Allan deviation of the resonant frequency at this mode is calculated to estimate the stability of the output frequency. The MEMS resonator is integrated into a low-noise phase-locked loop circuit using the HF2LI lock-in amplifier to track the mechanical resonance frequency continuously. The output frequency is logged on a PC as a time series for further analysis [64].

## 3. Theory and Modelling of the MEMS Resonator

**Figure 15:**
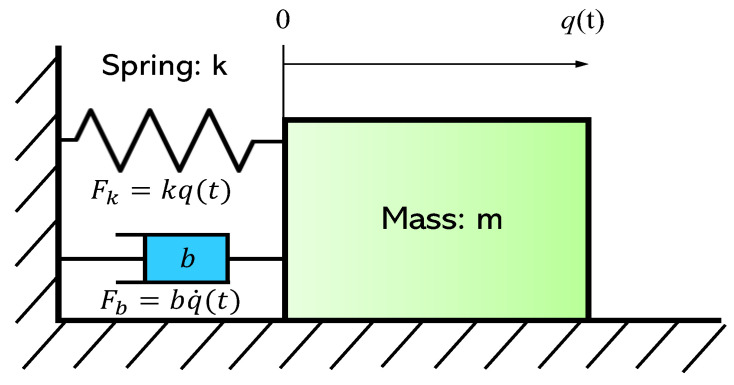
Mass spring damper representation of the MEMS resonator.

The relationship between the resonant frequency and mass sensitivity is stated in this section, considering the requirements of a MEMS sensor to detect the smallest mass possible. The mechanical resonance is modelled by a mass spring damper system in which the mass *m* attached to a linear spring of stiffness *k* oscillates, as shown in Figure 15. In practice, energy dissipation is introduced to the system by adding a damping term *b*.

Therefore, in the undamped case, a resonant MEMS sensor is simply a harmonic oscillator with the resonant frequency $f_{0}\;$ given by:

(2) $$f_{0}=\frac{1}{2\pi }\sqrt{\frac{k}{m}}$$

where k is the stiffness of the sensor, and m is the mass of the sensor [65]. By adding a small amount of mass Δm to the MEMS sensor, the frequency shift corresponding to the mass change is approximated to the first order and is expressed as:

(3) $$\Delta f=-\frac{1}{2}\frac{\Delta m}{m}f_{0}$$

In other words, knowing the frequency before and after mass addition determines the amount of mass (ultrafine particles, in this case) added to the MEMS sensor. Another key metric of the MEMS sensor is the quality factor which is given by:

(4) $$Q=\frac{f_{0}}{\delta f}$$

where δf is the half-power bandwidth, and $f_{0}$ is the resonant frequency. The quality factor determines the frequency shift resolution and hence the minimum detectable mass on the resonator surface.

The resonant frequency of the MEMS sensor is impacted by noise processes and environmental conditions due to temperature [66]. The Allan deviation provides a quantifiable measure of the frequency stability of the MEMS sensor under such conditions [67]. The equation that relates the minimum detectable mass and Allan deviation of the output frequency is given by:

(5) $$\sigma_{\delta M,f}=\frac{\sigma_{f}}{\left(\frac{\Delta f}{\Delta m}\right)}$$

where $\sigma_{f}$ is the Allan deviation of the MEMS oscillator output frequency for a given integration time, and Δf/Δm is the frequency-to-mass sensitivity [7].

## 4. Results

The results section describes the experimental results obtained for the sensitivity analysis and stability analysis of the MEMS resonator for silver nanoparticle deposition real-time monitoring and indoor particle deposition real-time monitoring, respectively.

### 4.1. Sensitivity Analysis

This section presents the experimental results of real-time monitoring of the resonant frequency during 50 nm silver nanoparticle deposition and unsized indoor particle deposition.

#### 4.1.1. Silver Nanoparticle Deposition

**Figure 16:**
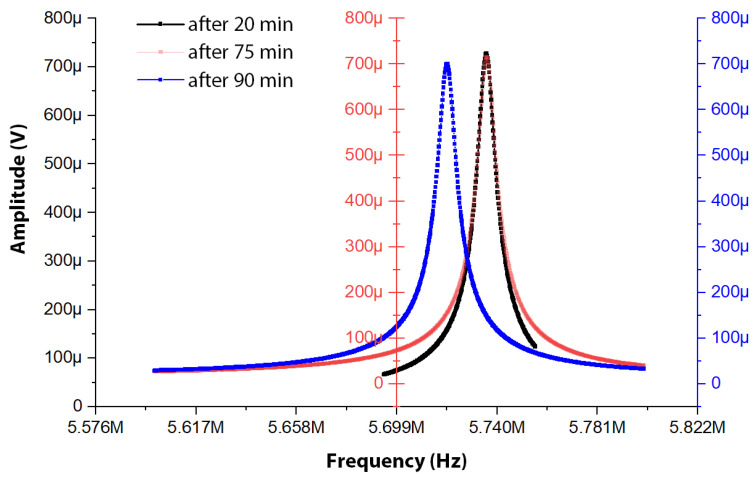
Open-loop frequency sweep response of the MEMS resonator after certain periods of silver nanoparticle deposition on the MEMS resonator surface.

The experimental results depicted in Figure 16 represent the open-loop frequency sweep responses of the MEMS resonator after certain time intervals of deposition as described in Section 2.2.1.

The resonant frequency change and the Q factor change of the MEMS resonator corresponding to the frequency response illustrated in Figure 16 is elaborated in Table 3.

**Figure 17:**
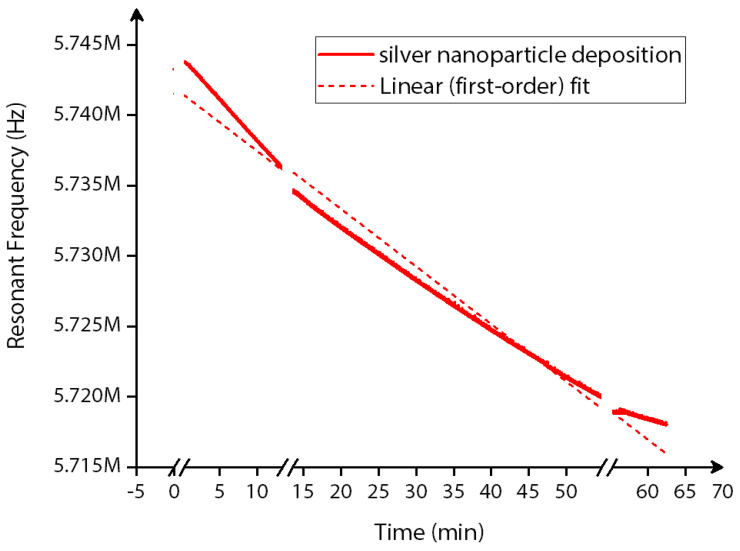
Real−time resonant frequency monitoring data for the silver nanoparticle deposition on the MEMS resonator surface. The piecewise linear fit function is used for this real-time resonant frequency monitoring data.

Similarly, the real-time monitoring of the silver nanoparticle deposition is plotted in Figure 17 for the entire time duration of deposition with a few time intervals in between. Throughout the entire time of deposition, a decrease in frequency shift is observed, with discretely stepped frequency shifts tracked by the phase-locked loop of the HF2LI lock-in amplifier.

#### 4.1.2. Indoor Particle Deposition

**Figure 18:**
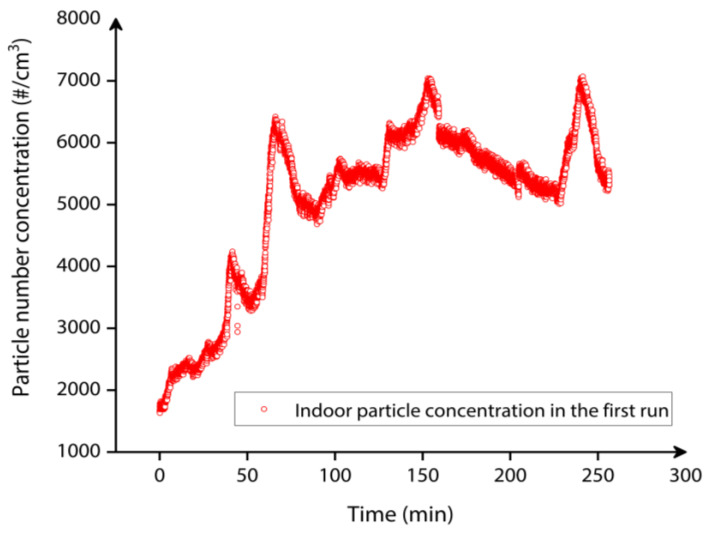
Indoor particle concentration monitored by the CPC reference instrument for approximately 4.27 h in the first set of indoor particle deposition experiment.

As explained in Section 2.2.2, Figure 18 highlights the indoor particle concentration monitored by the CPC reference instrument for approximately 4.27 h in the first set of indoor particle deposition with T-shaped tubing arrangement.

**Figure 19:**
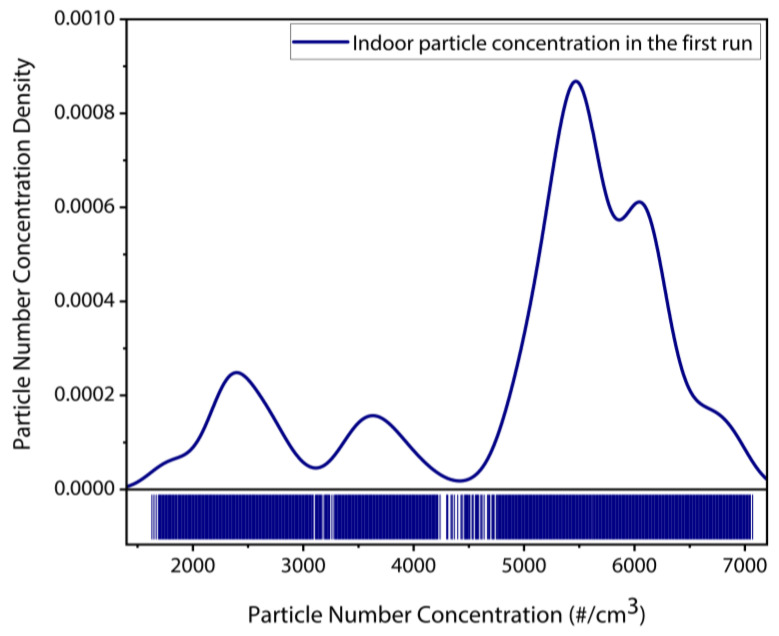
Particle number concentration distribution of the indoor particles monitored by the CPC reference instrument in the first set of deposition experiment.

Figure 18 demonstrates that the indoor particle concentration has nearly stabilized after initial 1.5 h (after 90 min), after which the vacuum pump was activated to draw the indoor particles towards the MEMS resonator through inertial impaction. Figure 19 depicts the particle number concentration distribution plot over the entire period that the CPC monitored the indoor particle concentration. This figure highlights that the maximum density of particle number concentration approximately 5000–6000 particles/cm^3^ is typically measured over the collection period. This, in turn, demonstrates that sufficient indoor particles were available for both impaction/collection on the MEMS resonator and measurement by the MEMS resonator.

**Figure 20:**
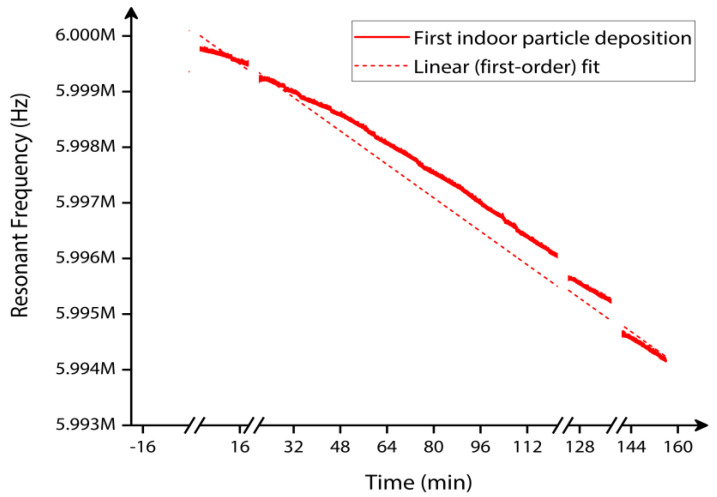
Indoor particle mass measured by the MEMS resonator over time in the first set of indoor particle deposition experiment.

The indoor particle mass monitored by the MEMS resonator based on resonant frequency shift is shown in Figure 20. In this experiment, the results corresponding to the frequency shift of the MEMS resonator are plotted, elucidating the resonant frequency change over the entire period of indoor particle deposition.

In this first set of indoor particle deposition experiments, the data collected from time t = 0 min until time t = 156 min were analyzed.

**Figure 21:**
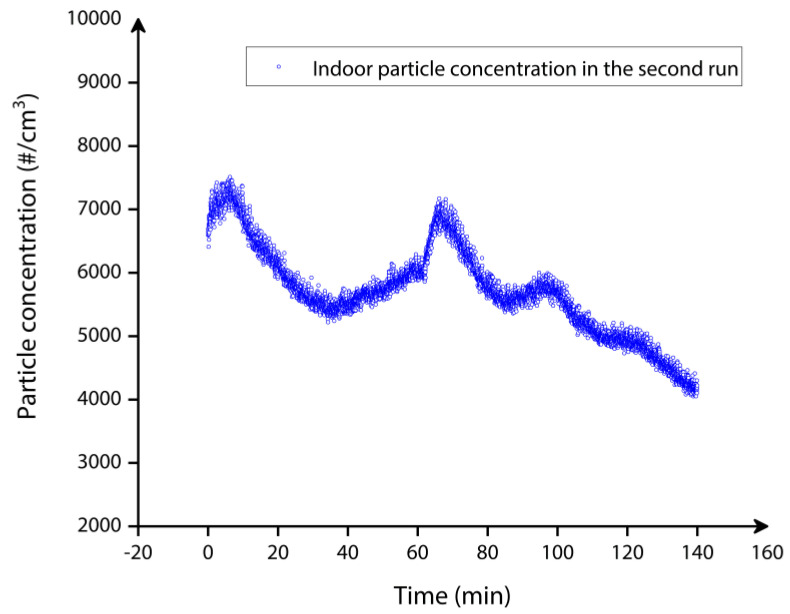
Indoor particle concentration monitored by the CPC reference instrument for approximately 2 h in the second set of deposition experiment.

**Figure 22:**
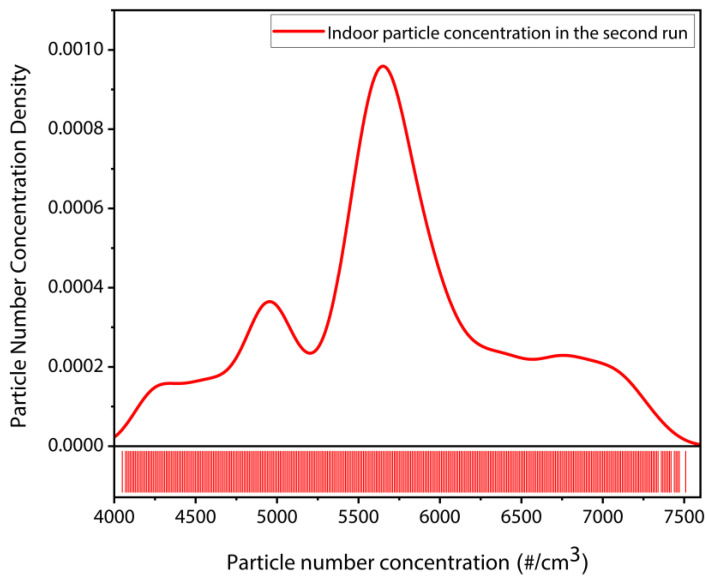
Particle number concentration distribution of the indoor particles monitored by the CPC reference instrument.

In the second set of indoor particle deposition experiment without the T-shaped tubing arrangement, the particle number concentration was recorded by the CPC reference instrument independently. The CPC data demonstrated in Figure 21 and Figure 22 indicate the indoor particle concentration monitored for the entire time duration of 2 h, in the second set of indoor particle deposition experiments. It can be seen from Figure 22 that the indoor particle concentration recorded by the CPC in the second set is equivalent to the indoor environment particle concentration recorded for the first set of indoor particle deposition experiment.

**Figure 23:**
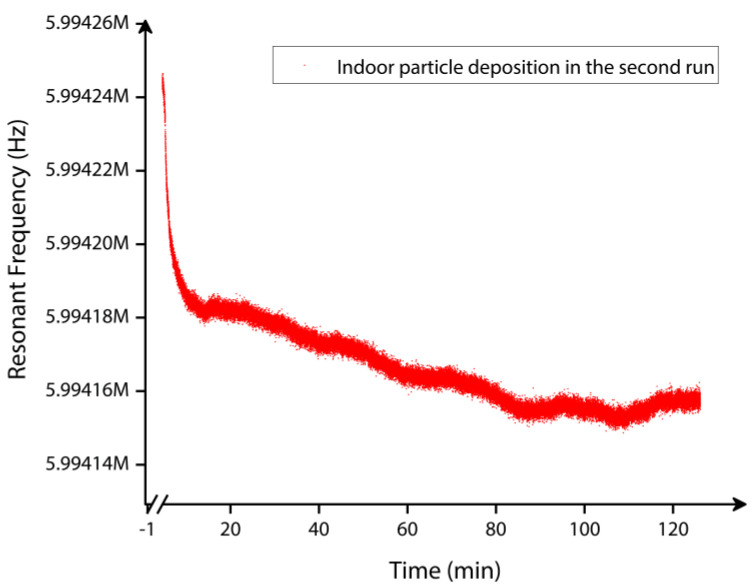
Indoor particle mass measured by the MEMS resonator over time (t = 2 h) in the second set of deposition experiment.

The MEMS resonant frequency shift data corresponding to the second set of indoor particle deposition experiment is plotted in Figure 23. For approximately two hours, the resonant frequency of approximately 5.99 MHz was tracked in this experiment while the CPC and MEMS resonator independently monitored the indoor particle concentration. The corresponding data indicate that the frequency shift is not significant in this scenario considered without the T-shaped tubing arrangement. Therefore, the number of indoor particles deposited onto the MEMS resonator is significantly lower compared to the previous scenario with the T-shaped tubing arrangement.

#### 4.1.3. The Frequency Stability Experiment

**Figure 24:**
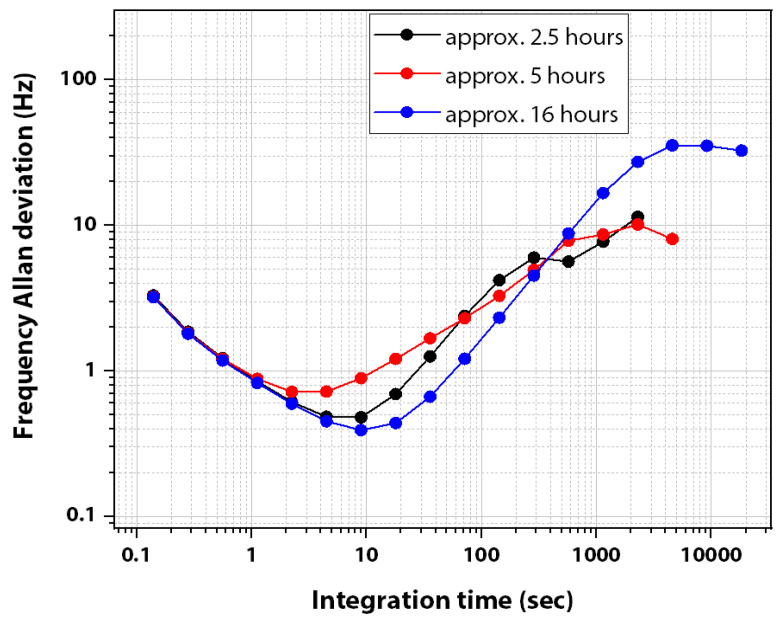
Frequency stability data for the 200 µm side-length square-plate MEMS resonator by setting the time constant to 0.1423 s (142.3 milliseconds). Data collection was performed at different time intervals by collecting 7.027 samples per second.

This section reports the results of the MEMS resonator frequency stability experiments for which the data is obtained in a closed loop for different time intervals such as 2.5, 5 and 16 h, approximately. The resonator frequency stability results demonstrated in Figure 24 indicate the Allan deviation of the resonant frequency at various time intervals.

The minimum Allan frequency deviation observed for a resonant frequency centered at approximately 5.99 MHz from the frequency stability plots depicted in Figure 24 is summarized in Table 4.

**Figure 25:**
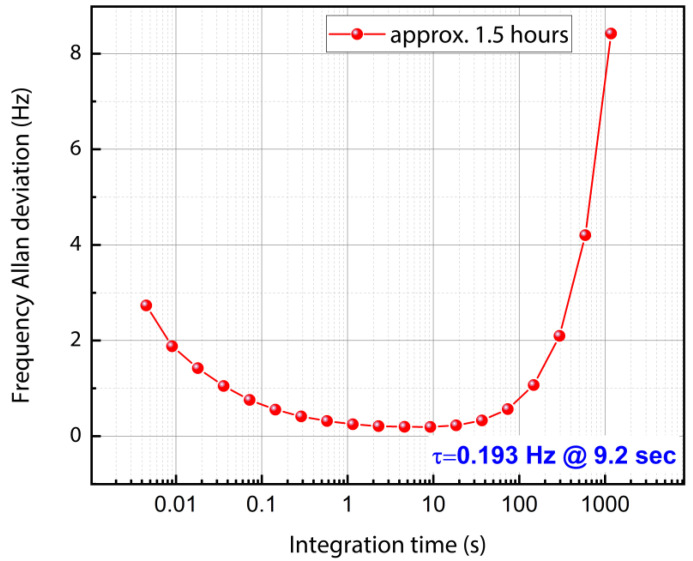
Frequency stability data for the 200 µm side-length square-plate MEMS resonator by setting the time constant to 0.00446 s (4.46 milliseconds). Data collection was performed for 1.5 h by collecting 224.9 samples per second.

The frequency stability was also observed by varying the sample rate to 224.9 samples/s for a time duration of approximately 1.5 h. The minimum Allan deviation as seen in the plot depicted in Figure 25 for this case is 0.193 Hz at τ = 9.2 s.

Based on the best-case Allan deviation data obtained by monitoring the resonant frequency in a closed-loop arrangement for 1.5 h with a time constant of 0.004 s (4 ms), the frequency resolution can be estimated to be **32.17 ppb**.

## 5. Discussion

### 5.1. Total Mass Estimation by the MEMS Resonator

In this section, the total mass accumulated on the MEMS resonator surface for both silver nanoparticle deposition and indoor particle deposition is estimated. Equation (3) is used to calculate the total amount of silver nanoparticles mass collected on the MEMS resonator surface. For a given resonator mass of 0.931 micrograms, the resonant frequency shift observed for the entire time duration of silver nanoparticle deposition is approximately 25,737.94 Hz. Depending on the corresponding frequency shift, the silver nanoparticles mass deposited on the MEMS resonator for time t = 55.29 min is estimated as 7.993 nanograms. Similarly, the indoor particle mass deposited on the MEMS resonator for time t = 156 min in the first set of indoor particle deposition experiments with T-shaped tubing arrangement depending on the frequency shift is estimated to be 1.732 nanograms. The corresponding frequency shift of the MEMS resonator observed in this case is approximately 5578.49 Hz. Having considered the first set of indoor particle deposition experiments and the total mass estimated, we will now consider the second set of indoor particle deposition experiments without the T-shaped tubing arrangement.

In the second set of experiment, the indoor particle mass deposited onto the MEMS resonator for time t = 2 h is estimated as 26.932 picograms for an observed frequency shift of 86.72 Hz. This, in turn, clearly demonstrates the impact of T-shaped tubing arrangement on the indoor particle deposition onto the MEMS resonator surface by highlighting the significant difference in frequency shift for the indoor particle mass added.

### 5.2. Minimum Mass Detection by the MEMS Resonator

This section calculates the minimum mass that can be detected by the MEMS resonator for both silver nanoparticle deposition and indoor particle deposition. However, depending on the Allan deviation obtained for different sample rates, Equation (5) is used to calculate the minimum detectable mass. The minimum Allan frequency deviation observed for the MEMS oscillator is calculated as 0.193 Hz as noted earlier.

Therefore, the minimum silver nanoparticles mass detectable by the MEMS resonator is estimated to be 60 femtograms using the minimum Allan deviation value. However, it should be noted that this mass estimation is for a measurement over a particular integration time and a particular value for another given integration time can be estimated from the plots provided in Figure 24 and Figure 25.

### 5.3. Comparison of MEMS Resonator Mass Estimation

Table 5 compares the mass sensitivity of the MEMS resonator described in this work to that of the other similar MEMS resonators reported in the literature.

## 6. Conclusions

This paper demonstrates a MEMS sensor arrangement for real-time monitoring of silver nanoparticle deposition and indoor particles with a view toward ultimately developing a portable setup for gravimetric sensing. A 5.999 MHz piezoelectric-on-silicon MEMS resonator is employed as the sensor element and is integrated within a MEMS Impactor Stage arrangement for testing ultrafine particulate detection. The experimental setup is elucidated in detail, with the assessment revealing the details of the setup around the MEMS element that can impact on the response. A total mass range of up to 7.993 nanograms and a minimum detectable mass limit ~60 femtograms to 0.12 picograms is measured depending on the sampling time and integration times chosen. While this paper provides evidence for the feasibility of applying MEMS resonators for particulate monitoring measurements, significant further work would be required to develop a miniaturized instrument that fully leverages the benefits of MEMS/electronics co-integration and further design optimization of the fluidics and particle deposition mechanism. The results hold promise that addressing these aforementioned engineering optimization tasks could establish the basis for a compact, portable and low-cost instrument for particulate monitoring.

## Figures and Tables

**Table 1:** Dimensions of the MEMS resonators.

Parameter	Value
Resonator area	$0.04\;{\mathrm{mm}}^{2}$
Resonator thickness	10 µm
Resonator mass	0.9316 µg
Resonator side length	200 µm
AlN film thickness	500 nm
Al/Cr electrode thickness	1 µm

**Table 2:** Extracted equivalent circuit MEMS resonator parameters.

Parameter	Value
$\mathrm{Resonant}\;\mathrm{Frequency},\;\omega_{m}$	5.99960621 MHz
$\mathrm{Motional}\;\mathrm{Resistance},\;R_{m}$	24.383 kΩ
$\mathrm{Motional}\;\mathrm{Inductance},\;L_{m}$	535.84 mH
$\mathrm{Motional}\;\mathrm{Capacitance},\;C_{m}$	1.3133 fF
Q factor, Q	828.42

**Table 3:** Resonant frequency and Q factor changes after certain periods of particle deposition.

Silver Nanoparticle Deposition	Resonant Frequency (MHz)	Q factor
After 20 min of silver nanoparticle deposition	5,735,573.28	813.374
After 75 min of silver nanoparticle deposition	5,735,774.76	813.403
After 90 min of silver nanoparticle deposition	5,719,478.28	790.794

**Table 4:** Allan deviation data for the frequency stability plots observed in Figure 21.

Frequency Stability Experiment	Allan Deviation
2.5 h (7.027 samples/second with a time constant of 0.14 s)	0.4796 Hz @ 8.96 s
5 h (7.027 samples/second with a time constant of 0.14 s)	0.7178 Hz @ 2.24 s
16 h (7.027 samples/second with a time constant of 0.14 s)	0.3921 Hz @ 8.96 s

**Table 5:** MEMS resonators utilized for sensing nanoparticles.

Ref.	Resonator	Resonant Frequency	Mass Sensitivity	Collection Mechanism	Transduction Mechanism	Readout Instrument
[59]	Silicon BAW square-plate resonator	3.1 MHz	29.5 Hz/ng	Inertial Impaction	Piezoelectric actuation, piezoelectric sensing	Frequency Counter
[68]	Silicon FBAR	1.6 GHz	2 μg/m^3^	Thermophoresis	Piezoelectric actuation and sensing	0.25 μm CMOS Circuit
[69]	Silicon I^2^-BAW resonator	2.87 MHz	0.02–0.4μg/m^3^	Inertial Impaction	Thermal actuation and piezoresistive sensing	Network Analyzer
[70]	Silicon resonant cantilever	43.92 kHz	8.33 Hz/ng	Dielectrophoresis	Piezoresistive actuation, piezoresistive sensing	Digital Multimeter, Spectrum Analyzer
[71]	Silicon resonant cantilever	43.92 kHz	10 Hz/ng	Electrostatic Precipitation	Piezoelectric actuation, piezoresistive sensing	Digital Multimeter, Spectrum Analyzer
[72]	Silicon resonant cantilever	221.5 kHz	36.51 Hz/ng	Electrostatic Precipitation	Piezoelectric actuation, piezoresistive sensing	Digital Multimeter, Spectrum Analyzer
[73]	Silicon BAW square-plate resonator	34.81 MHz	105.4 μm^2^/ng	Functionalized Surface Adsorption	Electrostatic actuation and sensing	Network Analyzer
[74]	Silicon FBAR	1.6 GHz	18 μg/m^3^	Thermophoresis	Piezoelectric actuation and sensing	Spectrum Analyzer
[75]	Silicon I^2^-BAW resonator	61 MHz and 20 MHz	1.6 kHz/pg	Inertial Impaction	Thermal actuation and piezoresistive sensing	Network Analyzer
[76]	Silicon I^2^-BAW resonator	2.5 to 5.5 MHz	5 Hz/pg to 42 Hz/pg	Inertial Impaction	Thermal actuation and piezoresistive sensing	Network Analyzer
**This work**	Silicon BAW resonator	5.999 MHz	59.94 fg to 0.12 pg	Inertial Impaction	Piezoelectric actuation and piezoelectric sensing	HF2LI Lock-In Amplifier

## Data Availability

No data were excluded from the analyses. The experiments were not randomized. The investigators were not blinded to allocation during experiments and outcome assessment.
